# Eye-tracking context formality effects in German and Japanese sentence processing

**DOI:** 10.1038/s41598-025-23609-4

**Published:** 2025-12-17

**Authors:** Valentina N. Pescuma, Kohei Haneda, Aine Ito, Katja Maquate, Pia Knoeferle

**Affiliations:** 1 Department of German Studies and Linguistics, Humboldt-Universität zu Berlin, Berlin, Germany; 2 Department of English, Linguistics and Theatre Studies, National University of Singapore, Singapore, Singapore; 3 Berlin School of Mind and Brain, Humboldt-Universität zu Berlin, Berlin, Germany; 4 Einstein Center for Neurosciences Berlin, Charité, Berlin, Germany

**Keywords:** Human behaviour, Social behaviour

## Abstract

Context information can rapidly affect language processing. Yet, whether this holds for social information like respect and formality (e.g., talking to a teacher vs. student), and across languages adhering differently to social hierarchy (German vs. Japanese) is unclear. We manipulated (in)congruence of formality (match vs. mismatch) in German and formality-style (matching or mismatching social respect) in Japanese. Participants encountered context-target sentence pairs (mis)matching in formality (German), or within-sentence formality-style (mis)match (Japanese). For Japanese, another factor ‘style’ (exalted vs. humble) created this mismatch (e.g., exalted style matches actions by a teacher but not student). For German, (in)congruence between subject and verb inflection served as a morphosyntactic baseline: we expected rapid effects. Longer first-pass times for mismatches than matches at the first mismatching word would indicate immediate formality processing. In German, formality mismatch effects emerged later, and in later measures, compared to first-pass morphosyntactic mismatch effects. In Japanese, formality-style effects emerged at the first mismatching word in a “late” measure (only for exalted style), in first fixation duration, post-verbally. Our results contribute to characterizing formality effects across languages that differ in their adherence to social hierarchy, and in which social markers in language are (Japanese) versus are not (German) part of grammar.

**Supplementary Information:**

The online version contains supplementary material available at 10.1038/s41598-025-23609-4

## Introduction

Psycholinguistic approaches to sentence comprehension have investigated what mechanisms and representations accommodate language users’ real-time language interpretation. Early theories such as^1^, for instance, described a ‘sausage machine’ (p. 291). A two-stage mechanism built a syntactic structure (a ‘parse’) without permitting influence from world knowledge on the initial parse. An alternative view assumed rapid feedback from higher-order expectations and world knowledge on initial syntactic processes. Evidence for its viability came from tasks in which participants rapidly - around 250 ms for the best - repeated (‘shadowed’) a speaker’s words^2^. Shadowing errors tended to not violate the speaker’s preceding syntax or semantics even at short shadowing lags, suggesting higher-level content influenced ongoing shadowing (comprehension and production). The extent and nature of top-down context effects on language processing continues to inspire current research (e.g.,^3–6^). In two eye-tracking experiments, we take up the topic of the time-course (delayed or immediate) and generality of context effects across languages for an under-investigated aspect of context - situation formality. Consider a formal situation in which a police director gives a lecture on a rivalry at the heart of riots. The language used would reflect the formality and status of the director. By contrast, if a statement referred to an ‘Olle’ (a German derogatory and colloquial term similar to English ‘old hag’), the situation conjured up in a reader’s mind would be considerably less formal. Would such context formality distinctions influence sentence processing at the earliest point in time?

Examining formality effects is further a means to assessing the generality of context effects on comprehension. Languages like German and Japanese differ in how they convey social information. Examining comprehension in Japanese gives us the possibility to gain insight into social context effects in a language that conveys honorific information like respect via grammar^35^ and in a culture that has been characterized as “vertically structured”^36^.The term ‘honorifics’ refers to respect conveyed in language. Honorific terms are known as *Keigo* (‘respectful language’) in Japanese. *Keigo* consists of three separate subclasses namely: *teinei-go* (‘polite style’), *sonkei-go* (‘exalted style’) and *kenjou-go* (‘humble style’). For instance, *mi-ru* (‘look’ [colloquial]), *mi-masu* (‘look’ [polite]), *goranni-naru* (‘look’ [exalted]), and *haikens-uru* (‘look’ [humble]) all refer to the action of looking; however, they cannot be used fully interchangeably, as each of them requires different socio-pragmatic contexts to appear in. The polite style is formed by attaching special morphemes to the verbal stem but the stem itself is shared with the colloquial style (see ^37^, p. 81, on details regarding the use of honorifics). Cui and colleagues^35^ highlight that for Japanese, honorific marking implicates “both socio-pragmatic and syntactic rules [...]. When speakers of lower social status use honorifics to address individuals of higher social status, humble or respectful verb forms must agree with the social property of the subject noun in a sentence”. Furthermore, studies on Korean^38,39^ highlight the syntactic nature of honorification, demonstrating that honorifics play a role in facilitating the resolution of null subjects.

Many aspects of world knowledge and context can rapidly influence comprehension and the resolution of local structural ambiguity, among them animacy^7^, plausibility^8^, thematic fit (e.g., a cop being a good agent for arresting a criminal^9^, and object arrangements^10,11^). The reach of lexical information, world knowledge and visual perception into the resolution of temporary structural ambiguity inspired accounts of sentence processing that accorded a central role to the lexicon (e.g.,^12^). It further supported immediate interaction of syntactic parsing with visual perception. Sentence processing accounts in the 1990s turned to fine-grained lexical and coarse-grained structural experience^13^ in accommodating comprehension (see also^9,14^). From the 1990s, accounts also considered probabilistic experience in parsing^15,16^, events and situations^17,18^, and visual context^19^. This was perhaps inspired by earlier research on modeling, such as work on language comprehension^20^ as the construction of a mental representation, i.e., a mental model, of what the utterance describes, or work on knowledge representation. Such research has evolved from structured *schemata*^21^ to more dynamic, connectionist models of learning via distributed representations^22^, up to the construction of situation models that integrate language and context^23^, and finally to grounding of lexical semantics in visual perception, via a link between linguistic representations and perceptual features of events^24^. Since then, empirical evidence (e.g.,^25–27^) and current models of real-time sentence processing have also foregrounded the importance of contextual information in the form of situations (e.g.,^28^). While even the non-linguistic visual context plays a role in accounts of sentence processing^4,10,29,30^, aspects such as the formality of a situation are under-investigated. Most of the reviewed accounts, indeed, accommodated comprehension in “average contexts”, without specifying situational-functional settings like formality (see ^31,32^, for integrating a notion of social context into accounts of language processing). However, in recent years, efforts have increasingly focused on deepening our understanding of situation-functional language use through the proxy of register variation. For instance, an acceptability judgment study by Rotter and Liu in 2024^33^ explored how register influences the use of negative concord and negative polarity items in English, highlighting the impact of situational context on linguistic choices. Similarly, a study by Wiese et al. in 2024^34^ examined register effects on language contact phenomena in Namibian German, showing how social meaning and orderly heterogeneity shape participants’ evaluations of linguistic forms across varying communicative contexts.

In the present work, we accordingly assessed to what extent formality is rapidly integrated during sentence processing in German and Japanese, respectively. The first study on German set out to replicate delayed effects of formality mismatch, conveyed via lexical variation, which were recently found in a similar study, relative to mismatch effects in subject-verb inflection. They are part of grammar and should thus be processed rapidly (see ^40^). We asked how formality (mis)matches between context and verb register impact real-time sentence processing and whether or not formality of a preceding linguistic context interacts with morphosyntactic mismatch, as indexed by subject-verb number agreement. By ‘register’, we mean “the conventionalized and recurrent linguistic patterns of (individuals in) a speech community depending on the situational-functional context”^41^. Given the natural sciences audience of the journal, we decided to use the term ‘formality congruence’ when talking about (in)congruence between the formality of the context and ensuing register in language. The second study on Japanese investigated within-sentence mismatches between formality characteristics of the morphosyntactic marking or lexical semantics of the verb, with a focus on socio-hierarchical context rather than on merely (as was the case for the study on German) situational formality. Assessing these incongruence effects for two levels of style (exalted vs. humble) further permitted us to explore biases in how formality congruence is processed. Mismatches compared with matches were expected to elicit more processing difficulty (see Analysis for an introduction of the dependent variables and the linking assumptions; see Operationalized hypotheses for detailed hypotheses).

## Methods

### Participants

In the German experiment, 43 participants, ages 18–31 (*M* age = 24, *N*(male) = 12) took part. Participants were all native speakers of German who reported not having learned any other languages before the age of 6. In the Japanese experiment, 37 participants took part, ages 18–31 (*M* age = 26, *N*(male) = 10). Participants were all native speakers of Japanese who reported not having proficiently learned any other languages before the age of 6. In both studies, participants had normal or corrected-to-normal vision. Ethics approval was given by the ethics board of the German association for linguistics (DGfS, #2019–07 A-200424). All research was performed in accordance with relevant guidelines and regulations, and written informed consent was obtained from all participants.

### Materials and design: German study

We created 32 critical items (see Table 1^40^). The final set of stimuli was based on formality ratings; web-based pretests were conducted to assess the formality of target and context sentences constituting the critical items (see Sect. 2.1.3 in^40^, for further details). Each item comprised two context sentences and one target sentence (German subject-verb-object order, e.g., *Der Polizist inhaftierte*^*formal*^ */ schnappte*^*informal*^ *die Aktivistin*, Engl. transl. ‘The policeman detained ^*formal*^/nabbed^*informal*^ the activist’). Context formality was conveyed by the context sentences (e.g., formal context: *Während der gestrigen Ausschreitungen waren die Einsatzkräfte gnadenlos. Die Polizeidirektorin referierte die Rivalität*:, Engl. transl. ‘During yesterday’s riots, the emergency forces were merciless. The police director lectured on the rivalry:’. Informal context: *Bei der Demo gestern war die Stimmung richtig heftig. Die Olle hetzte die Protestler*:, Engl. transl. ‘The atmosphere at the demo yesterday was really intense. The old hag stirred up the protesters:’). A 2 × 2 repeated measures design crossed *formality congruence* (match vs. mismatch of context formality and the verb in the target sentence) with *subject-verb congruence* (correctly inflected verb in third person singular vs. mismatching infinitive form violating subject-verb number agreement), yielding four conditions (see Table 1). Counterbalancing by context formality across lists yielded eight versions of each critical item. We created 8 base lists, each containing 32 critical items, interleaved with 56 filler items. Item-condition combinations were assigned to the lists using a Latin Square design, with order of items pseudo-randomized. Filler items also had two context sentences plus another sentence. 25% of fillers contained formality-register or subject-verb mismatches in one of the context sentences. This was done in order to prevent participants from discriminating critical from filler items. 75% of all filler items were followed by a “yes” / “no” comprehension question as an attention check. Stimuli, data and code are provided in our OSF repository.

### Materials and design: Japanese study

We created 32 critical items (see Table 1 for an example), which were previously normed via an acceptability study (see Supplement; Figures S1 and S2) as well as via a self-paced reading task (see Supplement; Table S1, Figures S3 and S4). Each critical item sentence introduced a subject associated with higher or lower social status (e.g., a teacher vs. a student). Morphological / lexical marking on the sentential verb provided further information regarding the expected degree of formality. The Japanese honorific verbs which we used were a mix of “lexically entrenched” honorific verbs (i.e., irregular or suppletive forms) and morphologically transparent honorific verbs (i.e., regular, predictable forms where honorification is explicitly marked with morphology). Thus, the stimuli contained both morphological and lexical manipulations. Out of the 32 critical items, 28 were morphologically marked and 4 were lexically marked. To create the stimuli, we first extracted the most frequent verbs from the Tsukuba Web Corpus (https://tsukubawebcorpus.jp/en/). We then converted each one of the 32 critical verbs into the corresponding exalted and humble forms by adding the morphological markings ’o(go)-V-ninaru’ and ’o(go)-V-suru’, respectively. When 4 verbs (*to say*,* to see*,* to go*,* and to come*) ended up sounding extremely unnatural with the above-mentioned morphological markings, we marked the honorification of these 4 verbs lexically. A 2 × 2 repeated measures design crossed the factors *formality-style congruence* (subject and verb honorifics congruous vs. incongruous) and *style* (exalted vs. humble), yielding four conditions (Table 1B). To illustrate the (in)congruence: The student is expected to use the exalted style when referring to actions performed by the teacher, but the humble style when referring to his/her own actions performed for/to the teacher. For instance, the following example (Table 1B sentence 1) is a formality-congruous sentence in which the exalted form of the verb ‘look’ *gorannina-tta-atode* is appropriately used to honor the subject: the teacher. On the other hand, the humble form of the verb ‘look’ *haikens-ita-atode* mismatches the formality-style expectations set up by mentioning the teacher, rendering the sentence pragmatically infelicitous. We constructed 56 filler items with a similar syntactic structure to the critical items, without any manipulation of formality-style congruence, 50% of which were grammatical and 50% ungrammatical. Critical items were assigned in a Latin Square design to 4 base lists. Items were pseudo-randomized for each participant. Stimuli, data and code are provided in our OSF repository.

### Procedure

For both studies, we recorded participants’ eye movements monocularly using an EyeLink 1000 Plus desktop mount eye-tracker (SR Research, Mississauga, Ontario, Canada). Prior to data acquisition, we performed tracker calibration and validation, with a nine-point calibration procedure. Following validation, values were accepted if the maximum error was smaller than or equal to 0.5 degrees of visual angle. Following each experimental block, calibration and validation were conducted again, with the option for additional re-calibration between trials if deemed necessary due to a gradual decline in tracking accuracy over time. Participants read the stimuli silently and were instructed to respond to occasional comprehension questions pressing “yes” and “no” buttons on the provided response box. A short practice round including two items was administered at the start of the experiment.

### German study

All experimental stimuli were displayed on four consecutive displays: After an initial fixation dot at the center of the screen, participants read the first context sentence, followed by the second context sentence, and finally the target sentence. They were directed to read as quickly and accurately as possible, and to proceed to the next sentence or trial at their own pace by pressing a button. Whenever comprehension questions appeared, participants were instructed to respond with either “Yes” or “No”. An 8000 ms time-out was set for each display containing sentences or questions. Overall, the experiment took approximately 40 min, incorporating three brief self-paced breaks between the four experimental blocks to reduce eye strain.

**Table 1:** Example critical target sentence for the German study (A) and the Japanese study (B). Analysis regions indicated by superscripts. For A, a linguistic context preceded the target sentence. Formal version: *Während der gestrigen Ausschreitungen waren die Einsatzkräfte gnadenlos. Die Polizeidirektorin referierte die Rivalität*, Literal Engl. transl. ‘During the yesterday’s riots, were the emergency forces merciless. The police director lectured the rivalry:’; Paraphrase: ‘During yesterday’s riots, the emergency forces were merciless. The police director lectured the rivalry:’. Informal version: *Bei der Demo gestern war die Stimmung richtig heftig. Die Olle hetzte die Protestler.*’, Literal Engl. transl. ‘At the demo yesterday was the mood really heated. The old hag stirred up the protesters:’; Paraphrase: ‘The mood at the demo yesterday was really heated. The old hag stirred up the protesters:’. For the Japanese example B, no prior linguistic context was presented; instead, formality-style congruence was manipulated within the target sentence.

Example A						
Condition	German stimulus					
Formality and subject-verb match	Der	Polizist	**inhaftierte** ^verb^	die	Aktivistin^post−verbal object^	
	‘The	policeman-NOM	incarcerated	the	activist-ACC.’	
Subject-verb mismatch	Der	Polizist	**inhaftieren** ^verb^	die	Aktivistin^post−verbal object^	
	*‘The	policeman	incarcerate	the	activist.’	
Formality mismatch	Der	Polizist	**schnappte** ^verb^	die	Aktivistin^post−verbal object^	
	‘The	policeman	nabbed	the	activist.’	
Formality and subject-verb mismatch	Der	Polizist	**schnappen** ^verb^	die	Aktivistin^post−verbal object^	
	*‘The	policeman	nab	the	activist.’	

Example B						
Condition	Japanese stimulus					
Formality-style match + exalted style	kenkyusitu-de	sensei-ga	jikken.kekka-o	**gorannina-tta-atode** ^verb^	mail-ga^post−verbal subject^	todoki-mas- ita
	lab-OBL	teacher-NOM	experimental.results -ACC	look(exalted)-PST-after	email-NOM	arrive-POL-PST
	Paraphrase: ‘After the teacher looked at the experimental results in the lab, an email arrived.’					
Formality-style match + humble style	kenkyusitu-de	watasi-ga	jikken.kekka-o	**haikens-ita-atode** ^verb^	mail-ga^post−verbal subject^	todoki-mas-ita
	lab-OBL	I-NOM	experimental.results -ACC	look(humble)-PST-after	email-NOM	arrive-POL-PST
	Paraphrase: ‘After I looked at the experimental results in the lab, an email arrived.’					
Formality-style mismatch + exalted style	*kenkyusitu-de	watasi-ga	jikken.kekka-o	**gorannina-tta-atode** ^verb^	mail-ga^post−verbal subject^	todoki-mas-ita
	lab-OBL	I-NOM	experimental.results -ACC	look(exalted)-PST-after	email-NOM	arrive-POL-PST
	Paraphrase: ‘After I looked at the experimental results in the lab, an email arrived.’					
Formality-style mismatch + humble style	*kenkyusitu-de	sensei-ga	jikken.kekka-o	**haikens-ita-atode** ^verb^	mail-ga^post−verbal subject^	todoki-mas-ita
	lab-OBL	teacher-NOM	experimental.results -ACC	look(humble)-PST-after	email-NOM	arrive-POL-PST
	Paraphrase: ‘After the teacher looked at the experimental results in the lab, an email arrived.’					

### Japanese study

All experimental stimuli were displayed on two consecutive displays: After the initial fixation dot at the center of the screen, participants read the target sentence with an 8000 ms time-out. They were instructed to read the sentence as quickly as possible and press the button once they were finished reading. Upon pressing the button, the corresponding comprehension question was displayed, to which participants were instructed to respond with either “Yes” or “No”. Overall, the experiment took approximately 45 min, incorporating one 5-minute break between the two experimental blocks to reduce eye strain.

### Analysis: German study

Due to insufficient accuracy in answering comprehension questions (*≤* 75%), the data from 3 participants was discarded and all analyses were conducted on the data from the remaining 40 participants. We pre-processed the eye-movement data in Data Viewer (SR Research) whereby fixations *<* 80 ms with a maximal distance of 0.5 degrees were merged together, and remaining fixations *<* 80 ms and *>* 1000 ms excluded. Analyses were conducted on two predefined areas of interest; we analyzed the verb, i.e., the critical region, and the post-verbal object, i.e., the post-critical / spillover region (see Table 1 superscript labels). We anticipated that effects might emerge up to the spillover region where, for example, processing costs can be observed following morphosyntactic mismatches (e.g., ^42^).

The dependent variables were three eye-tracking measures, indexing different stages of processing ^43–46^: *first pass* (the sum of the duration of all fixations on an interest region before leaving it, reflecting early stages of language processing), *regression path duration* (the sum of the duration of all fixations on an interest region and to its left before exiting it rightward, reflecting sentence integration processes), and *total time* (the sum of all fixation durations on an interest region, a cumulative and late measure reflecting post-lexical integration).

Before fitting any analysis models, we inspected the normality of the distribution of our data by means of a Q–Q plot, using the *qqnorm* function from the base R package *stats*^47^. As the data were not normally distributed, we ran a Box Cox test (using the function *boxCox* from the R package *car*^48^). Following the test, the three eye-tracking measures were log-transformed in order for the residuals to be approximately normally distributed. Linear mixed-effects models were fitted using *R*^47^ and the function *lmer* from the package *lme4*^49^. For visualization purposes, model estimates of the eye-tracking measures were then exponentially back-transformed for the scale to be more intelligible. The predictor term in each model comprised an interaction (and main effects) of *formality congruence* and *subject-verb congruence*. Model syntax initially featured a maximally complex random effects structure (RES), with the following by-participant and by-item intercepts and slopes:

lmer(log(first_pass) ~ formality_congruence*subject_verb_congruence + (1 + formality_congruence*subject_verb_congruence|participant) + (1 + formality_congruence*subject_verb_congruence|item).

Prior to significance testing of the fixed effects, the RES of each model was blindly assessed and, when necessary, reduced to a simpler structure (see ^50^) via inspection of the RES covariance matrix (using the function *VarCorr* from the R package *lme4*) and through a principal component analysis of the RES covariance matrix (via the function *rePCA* from the R package *lme4*). Random effects not (or only minimally) contributing to the variance of the model were thus simplified or removed. The model selection and comparison proceeded as follows: (1) Maximally-complex models were first fitted; (2) A rePCA analysis was performed to identify the level of contribution each component was making to the overall variance; (3) The random effect components that did not explain any substantial variance were removed; (4) A model comparison (via the *anova* function from base R *stats*) was performed to examine which of the two models (differing by one term) was a better fit; (5) 2–4 were applied iteratively until the most parsimonious fit was found (see ^50^).

### Analysis: Japanese study

Due to poor data quality, the data from 3 participants was discarded and all analyses were conducted on the data from the remaining 34 participants. As a sample size of *N* = 34 would not yield a balanced number of observations for each of the four base lists, analyses were also run with *N* = 32, which did not affect the pattern of results. Therefore, we consider data from all 34 participants in the analyses presented here. We used the same eye-movement data cleaning and pre-processing steps as for the German study in Data Viewer (SR Research). Analyses were conducted on three predefined areas of interest; we analyzed the subject noun, the verb and post-verbal subject noun phrase (see Table 1 superscript labels), using linear mixed-effects model (function *lmer* from the R package *lme4*). The predictor term in each model comprised an interaction (and main effects) of formality-style congruence (match vs. mismatch) and honorific style (exalted vs. humble). Model syntax initially featured a maximally complex random effects structure (RES), with the following by-participant and by-item intercepts and slopes:

lmer(log(first_pass) ~ formality_style_congruence*style + (1 + formality_style_congruence*style|participant) + (1 + formality_style_congruence*style|item).

The model selection and comparison proceeded as described for the German study. In the case of the Japanese study, this comparison procedure led, in all cases, to random-intercept-only models, e.g.,.:

lmer(log(first_pass) ~ formality_style_congruence*style + (1|item) + (1|participant)).

### Operationalized hypotheses

#### German study

Based on prior results, we expected effects of the subject-verb mismatch to appear rapidly during the verb or post-verbal object (e.g., ^51–55^) with longer reading times to mismatches than matches. The null hypothesis predicted no difference as a function of formality congruence. To the extent that inferences about formality from the context sentence rapidly inform semantic interpretation, however, we expected to see effects in first pass and / or regression path duration at the target verb, much like we expected for the subject-verb congruence manipulation. Effects, by contrast, in total times at the verb or in regression path duration and / or total times at the post-verbal object noun phrase would suggest that formality congruence effects are processed with some delay. To the extent that formality congruence influences the processing of subject-verb congruence, we expected to see a reliable interaction of the two manipulated factors in first pass and / or regression path duration at the target sentence verb, such as durations for subject-verb mismatches occurring alongside a formality mismatch effect. Delayed interaction effects might emerge either in total times at the verb or in regression path duration and / or total times at the post-verbal object noun phrase. Interaction effects would suggest the manipulated factors are not independent.

#### Japanese study

Assuming that the use of the Japanese honorifics is computed by L1 Japanese-speaking comprehenders in a similar manner as German register-formality is computed by L1 German-speaking comprehenders, similar results to those found in the main experiment of Pescuma et al.^40^ should be observed in the current study as well. That is, register-incongruent Japanese honorific stimuli will indeed impose more cognitive load compared to the register-congruent counterparts, but the effect is cumulative and delayed. In other words, it may only be observable on the post-verbal spillover region in the relatively late processing stages such as total reading times, and no reliable early or late effects may be observed on the critical verb region. At the same time, given the socio-pragmatic importance of honorifics in Japanese and the fact that honorific marking is grammaticalized, there is an intuitive possibility that the effects that will be observed in the present study might be more pronounced versions of those found in Pescuma et al.^40^; hence one could expect stronger late effects such as total reading times for the post-verbal spillover region or even on the critical verb region, or perhaps even earlier effects such as first-fixation durations or first-pass reading times for those regions. The null hypothesis predicted no difference as a function of formality-style congruence. The alternative hypotheses focused on teasing apart whether Japanese formality-style congruence is processed immediately much like other morphosyntactic agreement (see ^56^), who proposed Japanese formality-style agreement is derived via the same mechanism as the Agree operation commonly employed in Minimalist-style syntactic analyses^57^. Alternatively, formality-style processing and keeping track of socio-hierarchical relationships may occur delayed (see ^58^ for an example usage scenario). If formality-style congruence effects were part of the mechanism(s) responsible for morphosyntactic processing, then we would expect to see rapid effects in first-fixation durations and / or first-pass times at the first word at which the incongruence can be detected, the verb. Effects could alternatively emerge later (e.g., ^59^), for time-course differentiation) at the post-verbal subject, either in early measures, or in measures associated with “later” processes like total time. The latter outcome would suggest that formality-style effects are part of later, pragmatic, inferences. Initial hypotheses had also considered the possibility of increased second-pass effects for formality mismatches than matches to the pre-verbal subject noun phrase; we omit these in the results report due to potential confounds (lexical differences).

## Results

**Fig. 1:**
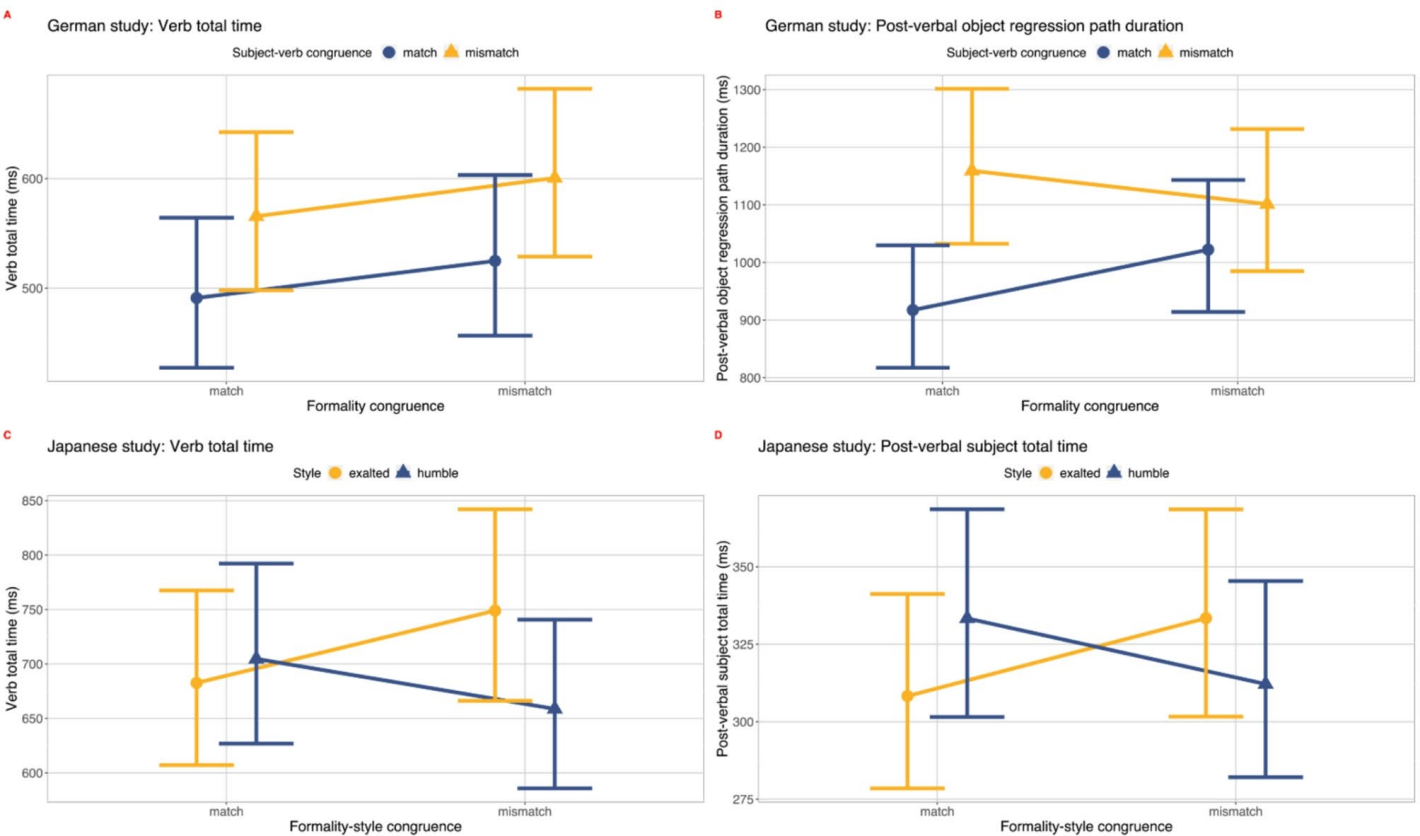
Illustration of formality congruence effects for the German (A-B) and of formality-style congruence for the Japanese (C-D) study.

Figure 1 showcases the observed formality and subject-verb congruence effects of the German study (Fig. 1A-B), and the formality-style congruence effects for the Japanese study (Fig. 1C-D).

### Inferential results: German study

We present the results of our analyses on first pass, regression path duration and total time for both regions of interest, verb and post-verbal object. Our analyses of the verb and post-verbal object showed similar patterns of results as previously reported^40^.

Analysis of first-pass and regression path at the **verb** did not reveal effects of formality or subject-verb congruence. Additionally, we analyzed an even earlier measure, first fixation duration, which revealed no effects of either formality-register or subject-verb congruence at the verb and post-verbal object regions. For each model listed below, in brackets are the predicted values for each condition (as obtained via the function *ggpredict* from R package *ggeffects*^60^.

Total time analysis of the verb (see Fig. 1A) revealed a main effect of subject-verb congruence (note that estimates in milliseconds are rounded up to the nearest whole number here and in all other analyses reported; mismatches: 600 ms [95% CI: 534, 673], *>* matches: 524 ms [95% CI: 466, 589]; *t* = 3.03, *p* = 0.005). A marginal effect of formality congruence emerged in total time at the verb (mismatches: 600 ms [95% CI: 534, 673]; matches: 565 ms [95% CI: 500, 637]; *t* = 1.73, *p* = 0.08).

First-pass analysis at the **post-verbal object** revealed effects of subject-verb congruence, albeit opposite to what was predicted, with longer first-pass times for matching (vs. mismatching) verbs (matches: 388 ms [95% CI: 347, 435] *>* mismatches: 353 ms [95% CI: 316, 393]; *t*=-3.18, *p* = 0.003). Regression path duration analysis at the post-verbal object showed a main effect of subject-verb congruence. In line with patterns of overt syntactic violations (e.g., ^54, 61^), regression path durations were longer for nouns following subject-verb mismatching (vs. matching) verbs (*t* = 4.77, *p* < 0.001; see Fig. 1B). Furthermore, the analysis revealed an interaction between subject-verb and formality congruence (*t*=-2.79, *p* = 0.005), with a larger difference in regression path durations following subject-verb mismatching (vs. matching) verbs in the formality match (vs. mismatch) condition (in subject-verb congruence mismatch condition, formality match: 1159 ms [95% CI: 1044, 1287] *>* formality mismatch: 1101 ms [95% CI: 996, 1217]; in subject-verb congruence match condition, formality mismatch 1022 ms [95% 921, 1134] *>* formality match 917 ms [95% CI: 820, 1026]; see Fig. 1B). Analysis of total time for the post-verbal object revealed only a trend towards an effect of formality congruence, in the predicted direction: Longer object total reading times were observed following formality-mismatching (vs. matching) verbs (mismatches: 649 ms [95% CI: 582, 724] *>* matches: 635 ms [95% CI: 567, 711]; *t* = 1.81, *p* = 0.07). Full model outputs are reported in the Supplement, Table S2.

### Inferential results: Japanese study

We present the results of the analyses conducted on first fixation duration, first pass time, regression path duration and total time for both regions of interest, verb and post-verbal subject. At the **verb**, no significant main effects of formality-style congruence emerged in any measure (all *t*s *<* 1.32). The 2 × 2 analysis revealed that this was the case due to an asymmetry in formality-style congruence effects for exalted compared with humble style: A trend towards a first-pass interaction of style and formality-style congruence emerged at the verb (*t*=-1.77, *p* = 0.08). First-pass differences (longer first pass for mismatches than matches) were observed in the exalted but not in the humble condition (in the exalted condition, formality-style mismatches: 489 ms [95% CI: 444, 540] *>* matches: 469 ms [95% CI: 427, 514]; in the humble condition, matches: 457 ms [95% CI: 415, 504] *>* mismatches: 427 ms [95% CI: 383, 475]). Total time analysis of the verb region (see Fig. 1C) revealed a significant interaction effect of style and formality-style congruence (*t*=-2.59, *p* = 0.01), with a greater increase of total time for exalted (vs. humble) style in the formality-style congruence mismatch (vs. match) condition (in the exalted condition, formality-style mismatches: 749 ms [95% CI: 672, 835] *>* matches: 683 ms [95% CI: 615, 758]; in the humble condition, matches: 705 ms [95% CI: 632, 786] *>* mismatches: 659 ms [95% CI: 586, 741]). We had no specific hypotheses about the main effect of style on eye movements during reading. For completeness sake, we report that a significant main effect of style emerged in first fixation duration analysis at the verb, with longer first fixation duration for humble (vs. exalted) style (humble: 226 ms [95% CI: 217, 236] *>* exalted: 214 ms [95% CI: 206, 222]; *t*=-2.77, *p* < 0.01).

First fixation duration analysis of the **post-verbal subject** revealed a significant main effect of style, with longer first fixations for exalted style (vs. humble, *t* = 2.12, *p* = 0.04), and a significant interaction effect of style and formality-style congruence (*t*=-2.01, *p* < 0.05), with an increase in first fixation duration for exalted (vs. humble) style only in the formality-style congruence mismatch condition (in the exalted condition, formality-style mismatches: 239 ms [95% CI: 226, 253] *>* matches: 222 ms [95% CI: 211, 233]; in the humble condition, matches: 221 ms [95% CI: 205, 233] *>* mismatches: 221 ms [95% CI: 209, 234]). First-pass and regression path duration analysis of the post-verbal subject revealed no effects of style or formality-style congruence (all *t*s *<* -1.42). Total time analysis of the post-verbal subject region (see Fig. 1D) revealed a significant interaction effect of style and formality-style congruence (*t*=-2.19, *p* = 0.03), with a greater increase of total time as a function of humble (vs. exalted) style in the formality-style congruence match (vs. mismatch) condition, and of exalted (vs. humble) style in the formality-style congruence mismatch (vs. match) condition (in the exalted condition, formality-style mismatches: 333 ms [95% CI: 305, 365] *>* matches: 308 ms [95% CI: 283, 335]; in the humble condition, matches: 333 ms [95% CI: 305, 365] *>* mismatches: 312 ms [95% CI: 282, 345]). Full model outputs are reported in the Supplement, Table S3.

## Discussion

In two eye-tracking reading experiments, we assessed effects of context formality on sentence processing by manipulating (in)congruence of formality (match vs. mismatch) in German and Japanese. Participants read context-target sentence pairs (mis)matching in formality (German), or sentences containing honorific mismatch between the pre-verbal subject and ensuing verb (Japanese). Would formality congruence effects resemble the rapid effects we see for subject-verb congruence, or rather somewhat delayed computations? Examining German and Japanese further permitted us to compare formality effects in languages that differ in their adherence to social hierarchy and in whether social marking (e.g., via honorifics) is morphologically encoded in the grammar, as it is in Japanese but not in German.

The observed eye-tracking pattern in the German study suggest that formality congruence effects occur later than rapid morpho-syntactic processing. Formality congruence effects occurred at a later region (post-verbal object) than subject-verb congruence effects (verb) and in later measures (total time and regression path duration post-verbally) than subject-verb congruence effects (first-pass time and regression path duration post-verbally).

In the Japanese study, clear formality-style congruence effects emerged at the verb in total time as evidenced by a significant interaction of formality-style congruence and style: formality-style congruence effects emerged as predicted (longer times for mismatches than matches) when the style was exalted. For humble style, formality-style matches took longer to process than mismatches. The marginal interaction of these factors also in first pass at the verb hints at the possibility of an even earlier effect. The reliable interaction of formality-style congruence with style in first fixation duration at the post-verbal subject corroborated the pattern observed at the verb and gives some more support to claiming early effects of formality-style congruence in Japanese. A caveat is in order, though, since for both analysis regions in the Japanese study, clear effects in first-pass time and regression-path duration were notably absent, perhaps calling in question the first-fixation duration results until further replication.

The reported findings suggest some delay in social context effects on sentence processing relative to the more rapid morphosyntactic processes. This claim is more robust for the German study, where both factors were manipulated independently, in contrast to the Japanese study, where both lexical and morphosyntactic factors were involved in the manipulation of social context. Nonetheless, these effects occurred incrementally during sentence processing, particularly at the verb and post-verbal regions that mismatched the preceding context in terms of formality (German) or honorific marking (Japanese). Notably, the only significant interaction in the German study occurred at the post-verbal region, where regression path duration was longer for subject-verb mismatches compared to matches, but only in the formality match condition. This finding contrasts with the prediction that a similar pattern would more strongly emerge in the formality mismatch condition. It also suggests a relatively slow and late unfolding of the effect of formality. Interestingly, the reversal of such effect occurred at an integration stage (regression path duration), suggesting that participants may have attempted to resolve morphosyntactic mismatches only when formality was congruent. This might imply that formality plays a role in “licensing” grammatical mistakes (see recent findings from ^62^, for example) or functions as a filter: when a formality mismatch occurs, further processing - including the detection of other mismatches – may become less in-depth.

Given these findings, particularly the delayed nature of formality effects, it follows that accounts of sentence processing may want to distinguish between formality and subject-verb congruence effects in their temporal integration. In our study, formality congruence was manipulated slightly differently across languages: In German, the linguistic register of the verb in the main clause matched or mismatched that of the context sentences, whereas in Japanese, honorific marking is part of grammar, resulting in “a more local” manipulation of formality. This distinction suggests that social context effects may need to be modeled differently depending on whether formality/honorific marking is grammaticalized (Japanese) or not (German). The fact that honorific marking is grammaticalized in Japanese may thus imply that formality processing unfolds along a different time course, and perhaps across different stages, than in German.

These results highlight the need for further investigation into how top-down processing and global context may influence sentence processing across different languages and situations. More specifically, our study suggests the possibility of manipulating formality in Japanese by creating a contextual cue that evokes an extremely formal mental representation, followed by a sentence where nouns and verbs either match or mismatch its formality (for instance, a contextual cue such as: “Last Sunday, a group of protestors condemning a politician’s corruption gathered in front of the Parliament building, and shouts of anger filled the air.” could be followed by a sentence where the lexical formality level (of both nouns and verbs) either matches or mismatches the previously given context).

## Supplementary Information

Below is the link to the electronic supplementary material.

Supplementary Material 1

## Data Availability

Supporting data, stimuli and code for the analyses reported in this article can be found in the project’s [OSF repository](https:/osf.io/27ect) .
