# Supplementary material for: Eye-tracking context formality effects in German and Japanese sentence processing
Source: Sci Rep. 2025 Dec 17;15:44030. doi: 10.1038/s41598-025-23609-4 (PMC12711875; doi:10.1038/s41598-025-23609-4)
Supplement: Supplementary file 1 — Supplementary Material 1 [file 41598_2025_23609_MOESM1_ESM.docx]

Supplementary material

August 20, 2025

In the supplementary material we provide: 1. A description of the norming tasks for the Japanese study (Acceptability rating study and Self-paced reading task) and the relative descriptive statistics;

2. Model outputs for the inferential statistics carried out in the analysis of the German and Japanese eye-tracking studies.

# Japanese study: Norming of stimuli

Experimental items were normed via an acceptability study and a self-paced moving-window reading task, both programmed with PCIbex and hosted on Clickworker for data collection.

## Acceptability rating study

Thirty-three self-proclaimed L1 Japanese speakers, aged between 18 and 31, were recruited for the study. Based on their responses to the post-experimental questions, one participant was judged to be a non-native speaker by the experimenter; the data were thus discarded. Data from 32 participants (*M* (age)=26.6, *N* (male)=20) were analyzed. 32 critical items in a 2 (*style*: exalted vs. humble) x 2 (*formality-style congruence*: match vs. mismatch) design, and 56 filler items with half of them grammatical and the rest containing grammatical errors in the form of a wrong case- marking particle attached to the subject noun. Based upon the 32 critical items, 4 Latin Square base lists were created, which were then interspersed with 56 filler items in multiple different pseudo- randomized orders in such a way that there was at least 1 filler item between two critical items. Each participant was presented with a list of 88 items (32 critical + 56 filler), one item at a time. Upon pressing the spacebar to signify completion of reading, a scale of 0-50 (with 0 being “the least likely” and 50 being “the most likely”) was subsequently displayed along with the question “How likely or unlikely do you think it is for a native Japanese speaker to utter this sentence?”. The acceptability rating study took approximately 10 minutes to complete.

### Descriptive statistics

Mean acceptability rating values were 28 (*SD* =15) in the exalted-match, 12 (*SD* =14) in the exalted- mismatch, 20 (*SD* =18) in the humble-match, and 23 (*SD* =16), in the humble-mismatch condition. Humble-mismatch was judged to be more acceptable than humble-match but for the exalted style, match (28/50) was judged more acceptable than mismatch (12/50). See Figures [S1](#_bookmark0) and [S2](#_bookmark5) for average ratings by condition and by item, respectively.


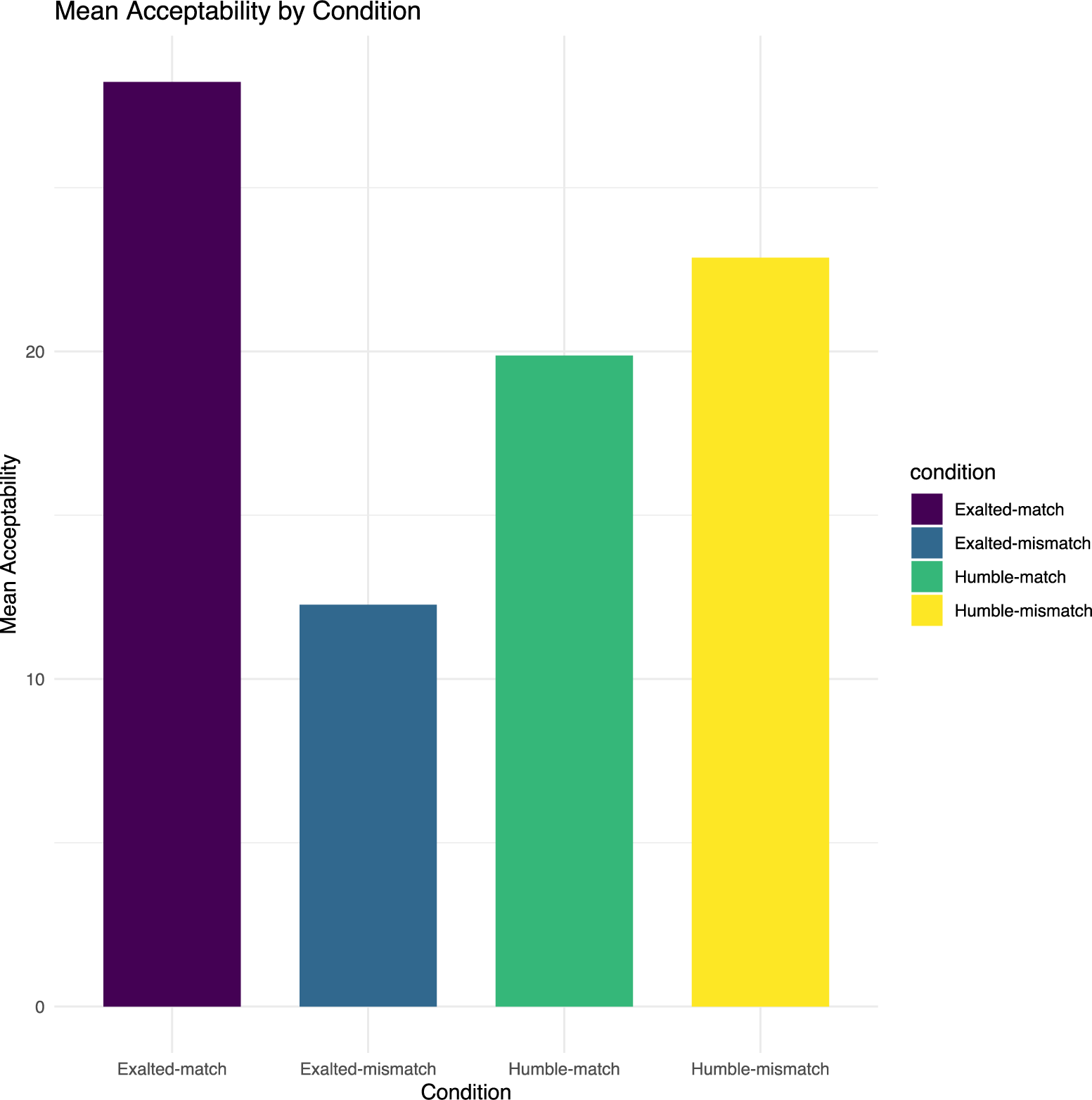


Figure S1: Illustration of mean acceptability ratings by condition for the Japanese study.

## Self-paced reading task

Thirty-three self-proclaimed L1 Japanese speakers, aged between 18 and 31, were recruited for the study. Their residential information was not made available but they could have been located anywhere in the world. Based upon the score of comprehension questions, 2 participants scored below the minimum passing criteria of 75%; their data were discarded. Data from 31 participants (*M* (age)=24.7, *N* (male)=21) were finally analyzed. [1](#_bookmark2) As in the acceptability study, each participant was presented with a list of 88 items (32 critical + 56 filler), one item at a time. Each item was segmented into six constituency chunks, namely: 1. adjunctive-adverbial region, 2. main-subject region, 3. main-object region, 4. main-verb region, 5. second-subject region, and 6. second- verb region. We adopted a moving-window paradigm, in which participants were instructed to press the spacebar to request a new sentence chunk to appear on the screen. Every time the subsequent sentence chunk appeared, the previously displayed chunk disappeared, hence only one sentence chunk was displayed on the screen at a time. Upon completion of reading each sentence, a comprehension question about the sentence was subsequently displayed on the screen, to which participants were instructed to respond with either “Yes” or “No” by pressing “F” or “J”. This ensured that participants stayed focused on the task and read each item properly (for an illustration of the procedure, see Figure [S3).](#_bookmark6) The self-paced reading tasl took approximately 20 minutes to complete.

### Descriptive statistics

Descriptive statistics for reading times of the verb and post-verbal regions are provided in Table [S1.](#_bookmark1) These are graphically presented in Figure [S4.](#_bookmark7)

Table S1: Reading times for the Japanese self-paced reading task, by condition and by region.

| **Condition** | **Mean reading**  **time (ms)** | **SD**  **(ms)** | **SE**  **(ms)** | **Region** |
| --- | --- | --- | --- | --- |
| Exalted-match | 731.64 | 450.57 | 28.61 | verb |
| Exalted-mismatch | 748.15 | 444.62 | 28.23 | verb |
| Humble-match | 707.86 | 445.92 | 28.32 | verb |
| Humble-mismatch | 699.68 | 468.62 | 29.76 | verb |
| Exalted-match | 504.96 | 218.26 | 13.86 | post-verbal  noun |
| Exalted-mismatch | 522.59 | 250.87 | 15.93 | post-verbal  noun |
| Humble-match | 538.49 | 263.17 | 16.71 | post-verbal  noun |
| Humble-mismatch | 542.74 | 306.58 | 19.47 | post-verbal  noun |

1Although only participants whose registered age fell between 18 and 31 were allowed to sign up for this experiment, four participants selected the option “above 31 years old” in the pre-experimental demographic questions. The data of these four participants were, however, not discarded, as the experimenter judged the registered information on the platform to be more accurate than their responses to the multiple-choice survey preceding the experiment.

# Linear mixed-effects model outputs

In this section we report, in the form of tables, the coefficients for the linear mixed models reported in the main text of the article.

## German study

Table [S2](#_bookmark3) contains the coefficients of the linear mixed-effects models performed to analyze the Ger- man eye-tracking data. The column names refer to the four models reported in the main text (Inferential Results: German Study): "Verb total time" refers to the total time analysis of the verb region, "Obj first pass" refers to the first-pass analysis of the post-verbal object region, "Obj regr path" refers to the regression path duration analysis of the post-verbal object region, and "Obj total time" refers to the total time analysis of the post-verbal object region. Please note that the estimates of the Intercept and of the predictors (formality congruence, subject-verb congruence, and the interaction of formality and subject-verb congruence) are expressed on a log scale, due to the transformation used in the models.

Table S2: Coefficients of the statistical models performed in the German eye-tracking study

|  | **Verb total time** | **Obj first pass** | **Obj regr path** | **Obj total time** |
| --- | --- | --- | --- | --- |
| (Intercept) | 6*.*30*^∗∗∗^* | 5*.*93*^∗∗∗^* | 6*.*95*^∗∗∗^* | 6*.*45*^∗∗∗^* |
|  | (0*.*06) | (0*.*05) | (0*.*05) | (0*.*06) |
| formality congruence | 0*.*03 | *−*0*.*02 | 0*.*01 | 0*.*03 |
|  | (0*.*02) | (0*.*02) | (0*.*02) | (0*.*02) |
| subj-verb congruence | 0*.*07*^∗∗^* | *−*0*.*06*^∗∗^* | 0*.*08*^∗∗∗^* | 0*.*02 |
|  | (0*.*02) | (0*.*02) | (0*.*01) | (0*.*02) |
| formality congruence:subj-verb congruence | *−*0*.*00 | 0*.*01 | *−*0*.*04*^∗∗^* | *−*0*.*02 |
|  | (0*.*02) | (0*.*02) | (0*.*01) | (0*.*02) |
| AIC | 2695*.*45 | 2326*.*33 | 2087*.*38 | 2322*.*87 |
| BIC | 2741*.*81 | 2372*.*67 | 2133*.*72 | 2369*.*24 |
| Log Likelihood | *−*1338*.*72 | *−*1154*.*17 | *−*1034*.*69 | *−*1152*.*44 |
| Num. obs. | 1276 | 1272 | 1272 | 1276 |
| Num. groups: participant | 40 | 40 | 40 | 40 |
| Num. groups: item | 32 | 32 | 32 | 32 |
| Var: participant(Intercept) | 0*.*05 | 0*.*07 | 0*.*06 | 0*.*06 |
| Var: item (Intercept) | 0*.*06 | 0*.*03 | 0*.*03 | 0*.*04 |
| Var: item subj-verb congruence | 0*.*01 |  |  | 0*.*00 |
| Cov: item (Intercept) subj-verb congruence | *−*0*.*01 |  |  | 0*.*00 |
| Var: Residual | 0*.*42 | 0*.*32 | 0*.*26 | 0*.*31 |
| Var: participant subj-verb congruence  Cov: participant(Intercept) subj-verb congruence Var: item formality congruence |  | 0*.*00  *−*0*.*00 | 0*.*00 |  |
| Cov: item (Intercept) formality congruence |  |  | *−*0*.*00 |  |
| *^∗∗∗^p <* 0*.*001; *^∗∗^p <* 0*.*01; *^∗^p <* 0*.*05 |  |  |  |  |

## Japanese study

Table [S3](#_bookmark4) contains the coefficients of the linear mixed-effects models performed to analyze the Japanese eye-tracking data. The column names refer to the four models reported in the main text (Inferential Results: Japanese Study): "Verb first pass" refers to the first-pass analysis of the verb region, "Verb total time" refers to the total time analysis of the verb region, "Subj first fix"

refers to the first fixation duration analysis of the post-verbal subject region, and "Subj total time" refers to the total time analysis of the post-verbal subject region. Please note that the estimates of the Intercept and of the predictors (formality-style congruence, style, and the interaction of formality-style congruence and style) are expressed on a log scale, due to the transformation used in the models.

Table S3: Coefficients of the statistical models performed in the Japanese eye-tracking study

|  | **Verb first pass** | **Verb total time** | **Subj first fix** | **Subj total time** |
| --- | --- | --- | --- | --- |
| (Intercept) | 6*.*13*^∗∗∗^* | 6*.*55*^∗∗∗^* | 5*.*42*^∗∗∗^* | 5*.*77*^∗∗∗^* |
|  | (0*.*05) | (0*.*05) | (0*.*03) | (0*.*04) |
| formality-style congruence | 0*.*01 | *−*0*.*01 | *−*0*.*02 | *−*0*.*00 |
|  | (0*.*02) | (0*.*02) | (0*.*01) | (0*.*02) |
| style | 0*.*04*^∗^* | 0*.*02 | 0*.*02*^∗^* | *−*0*.*00 |
| formality-style congruence:style | (0*.*02)  *−*0*.*03 | (0*.*02)  *−*0*.*04*^∗∗^* | (0*.*01)  *−*0*.*02*^∗^* | (0*.*02)  *−*0*.*04*^∗^* |
|  | (0*.*02) | (0*.*02) | (0*.*01) | (0*.*02) |
| AIC | 1637*.*66 | 1418*.*64 | 711*.*32 | 1462*.*63 |
| BIC | 1672*.*52 | 1453*.*51 | 745*.*21 | 1496*.*51 |
| Log Likelihood | *−*811*.*83 | *−*702*.*32 | *−*348*.*66 | *−*724*.*32 |
| Num. obs. | 1075 | 1075 | 935 | 935 |
| Num. groups: item | 128 | 128 | 128 | 128 |
| Num. groups: participant | 34 | 34 | 34 | 34 |
| Var: item (Intercept) | 0*.*00 | 0*.*01 | 0*.*00 | 0*.*00 |
| Var: participant (Intercept) | 0*.*07 | 0*.*09 | 0*.*02 | 0*.*05 |
| Var: Residual | 0*.*24 | 0*.*19 | 0*.*11 | 0*.*25 |
| *^∗∗∗^p <* 0*.*001; *^∗∗^p <* 0*.*01; *^∗^p <* 0*.*05 |  |  |  |  |


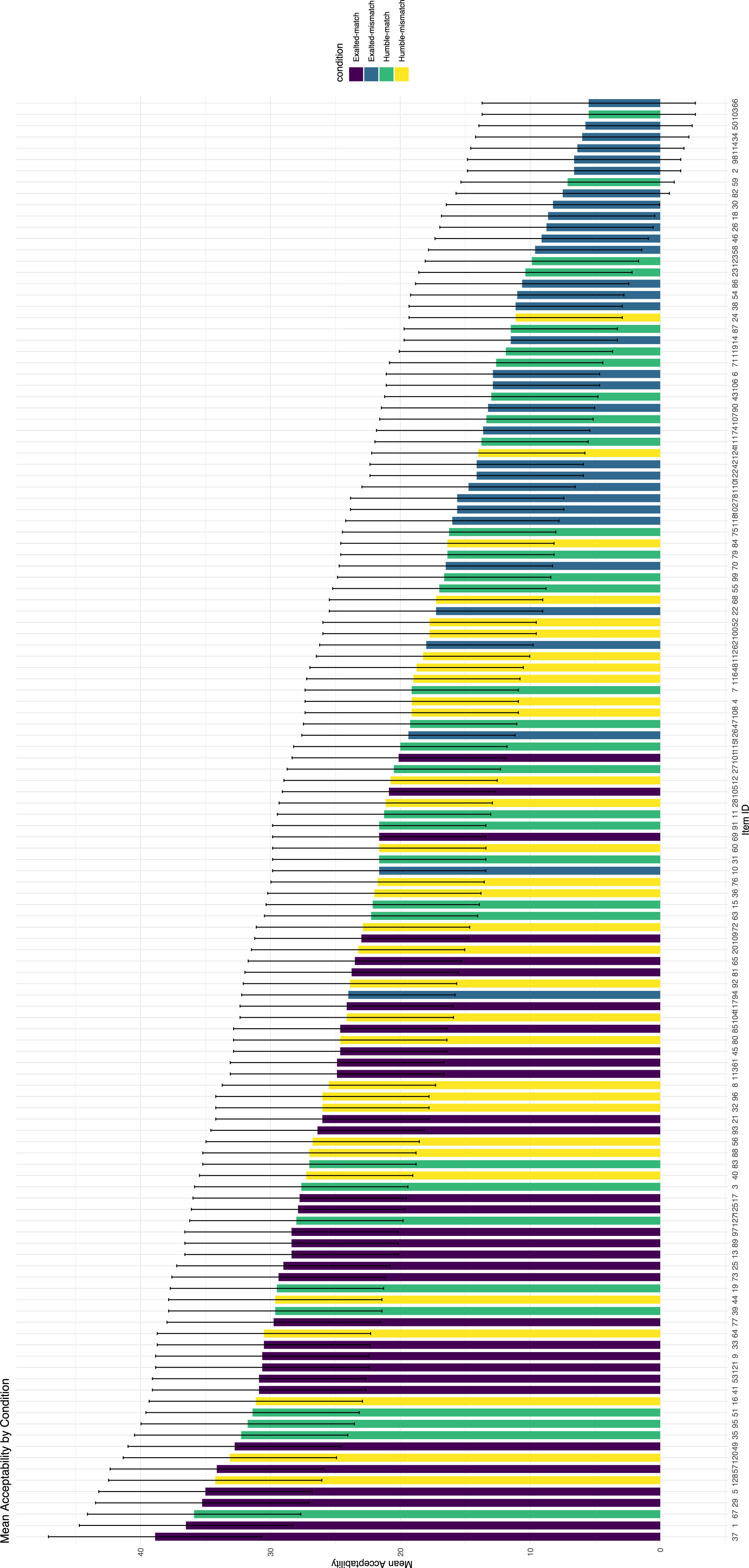


Figure S2: Illustration of mean acceptability ratings by item for the Japanese study.


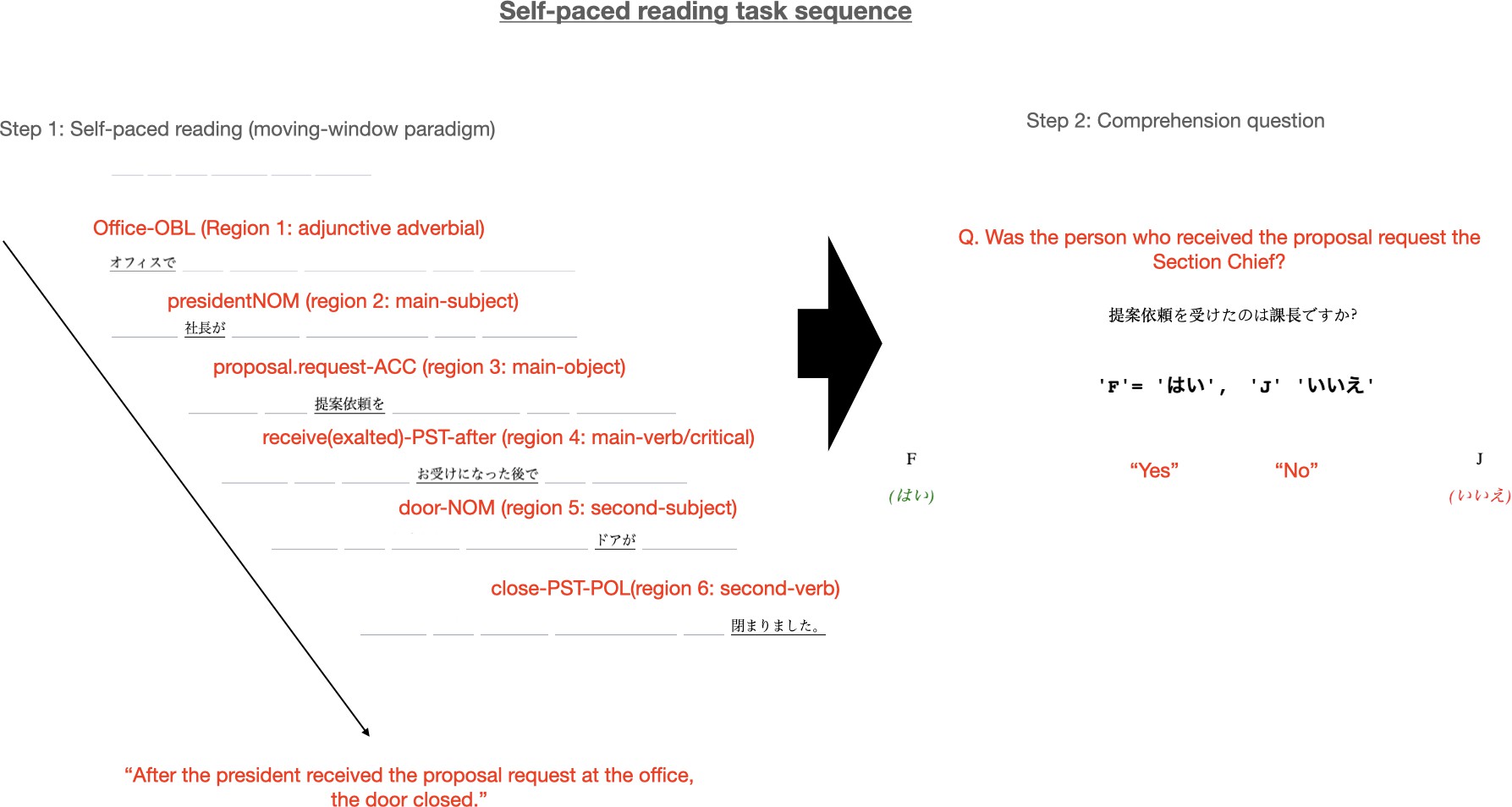


Figure S3: Illustration of the self-paced reading task, part of the norming procedure of the Japanese stimuli.


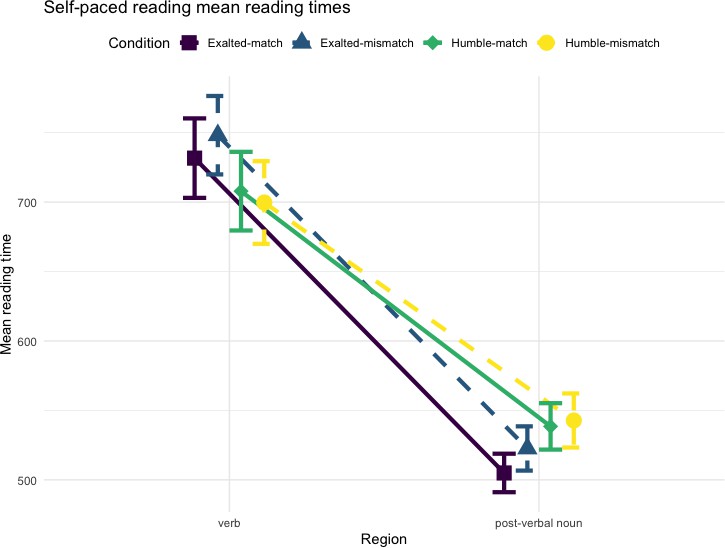


Figure S4: Illustration of reading times in the Japanese self-paced reading task by condition and by region.
